# Conventional and Emerging Counter Electrode Materials for Dye-Sensitized Solar Cells: A Comprehensive Review

**DOI:** 10.3390/ma19214693

**Published:** 2026-11-04

**Authors:** Md. Akhtaruzzaman, Abul Kashem Mahmud Hasan, Mohammad Ismail Hossain, Jean-Michel Nunzi, Md. Shahiduzzaman

**Affiliations:** 1 Department of Chemical Engineering, Faculty of Engineering, Islamic University of Madinah, Madinah 42351, Saudi Arabia; 2 Dhaka Solar Ltd., Dhaka 1212, Bangladesh; 3 Department of Electrical and Computer Engineering, University of California, Davis, CA 95616, USA; m.hossain.jub@gmail.com; 4 Department of Physics, Engineering Physics and Astronomy, Queens University, Kingston, ON K7L 3N6, Canada; nunzijm@queensu.ca; 5 Nanomaterials Research Institute, Kanazawa University, Kakuma, Kanazawa 920-1192, Japan

**Keywords:** dye-sensitized solar cells, counter electrodes, electrocatalysis, carbon-based materials, conductive polymers, transition metal compounds, photovoltaics

## Abstract

Dye-sensitized solar cells (DSSCs) are attractive photovoltaic devices due to their low manufacturing costs, simple processing steps, transparency and compatibility with flexible substrates. The counter electrode (CE) is vital for catalyzing redox reactions, thereby affecting charge-transfer efficiency and overall device performance. While platinum (Pt) remains the standard material for CEs, its high cost, limited availability and long-term chemical stability issues have driven research into more affordable alternatives. This review covers recent developments in both traditional and novel CE materials, including Pt-based compounds, carbon materials, conductive macromolecules, transition metal elements and subsequent composites. It compares their synthesis methods, structural and electrochemical properties, catalytic efficacy and photovoltaic performance. This paper concludes with an overview of recent encounters and future pathways, aiming to foster the progress of effective, durable and cost-saving CEs for next-generation DSSCs.

## 1. Introduction

The rapid use of fossil fuels has led to major concerns about resource depletion and environmental pollution, including greenhouse gas emissions and global warming [1,2,3]. As a result, there is a greater need for sustainable renewable energy sources. One of the most abundant and environmentally sound solutions is solar energy, as it can meet the world’s energy needs. There have been substantial improvements in photovoltaic (PV) technologies, and silicon-based photovoltaics are currently the most used in the market (~90%) for their stability, despite their high production cost [4,5,6]. Because of the problems of cost and scalability, alternative technologies such as dye-sensitized solar cells (DSSCs) have drawn significant attention. DSSCs have a few important advantages, namely, low cost, simple fabrication, transparency, tunable color, environmental friendliness and the possibility of flexible applications.

The study of photosensitivity began with Vogel, when the first ideas for DSSCs appeared at the end of the 19th century. It was not until almost a hundred years later that working DSSC devices were achieved, during which Gerischer and Tributsch showed early examples of DSSCs using chlorophyll (which is obtained from a tree leaf) as the photoactive material and ZnO as the photoanode [7,8,9]. DSSCs function on a principle similar to that of natural photosynthesis: just as plants convert solar energy into chemical energy, DSSCs convert light energy into electrical energy by means of photoinduced charge transfer [10]. Because ZnO-based systems have poor stability and efficiency, TiO_2_ has become a better photoanode material. Its suitable band alignment allows efficient injection of electrons from the excited dye, while its high transparency ensures maximum light absorption. Moreover, the mesoporous structure of TiO_2_ offers a larger effective area for dye molecule adsorption onto the photoanode and, thus, improves light absorption as well as the overall performance of the device [11,12,13,14].

In 1991, a major advance was achieved by O’Regan and Grätzel, who developed a pioneering device based on a TiO_2_ nanoparticle photoanode, a sensitizer (such as N719), and a liquid electrolyte, thereby establishing a semiconductor electrolyte junction. This device made it possible to achieve efficient light absorption and used a simple method of fabrication, and, for this reason, drew substantial interest in the field of photovoltaic research [15,16]. Unlike conventional p–n junction solar cells, it is the semiconductor electrolyte interface in a DSSC that is responsible for the built-in potential, and, as a result, there is no requirement for complex junction fabrication. Since then, continuous improvements in materials and device structure have led to further increases in the efficiency and stability of DSSCs [8,17].

**Figure 1:**
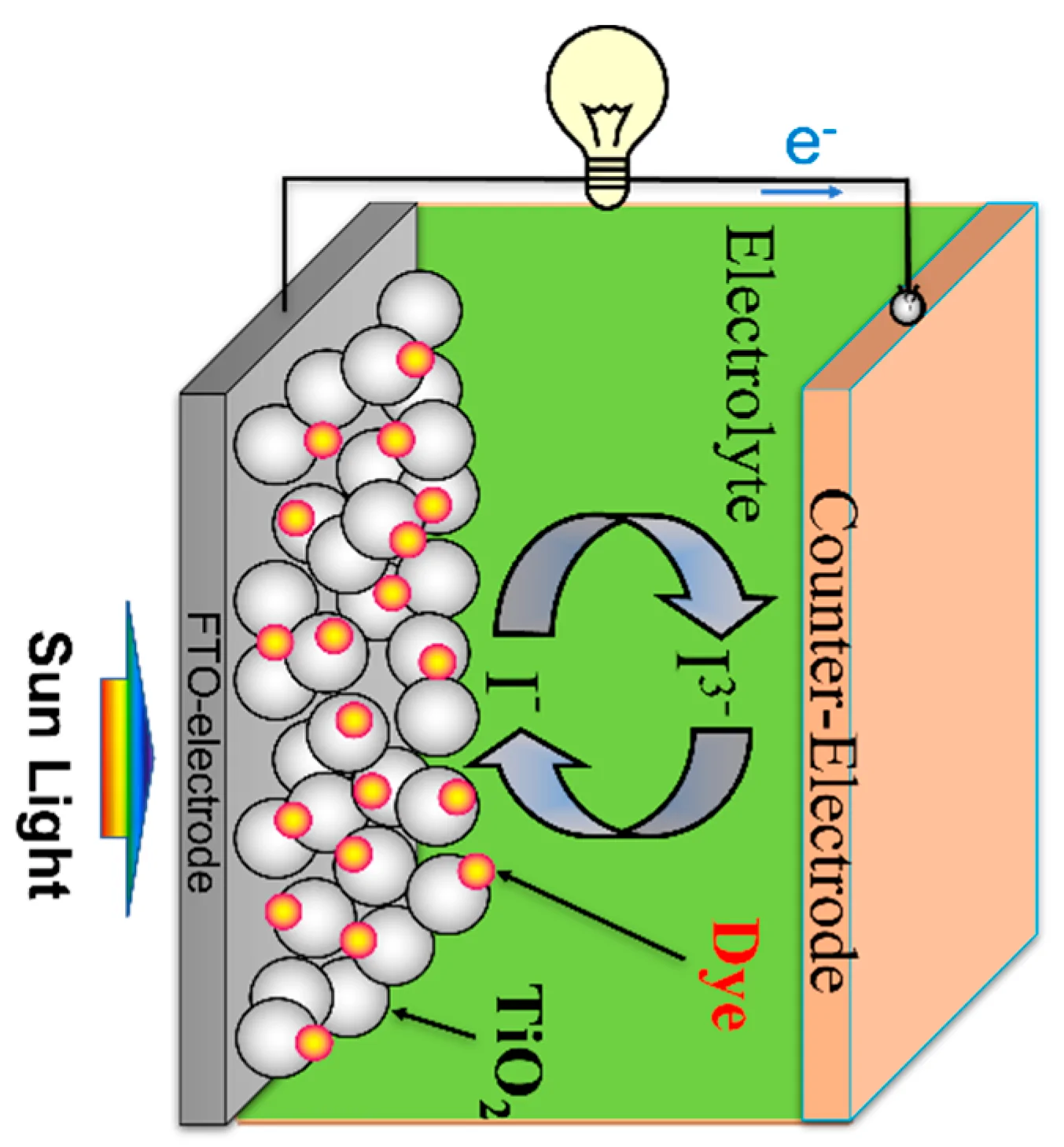
Schematic illustration of the DSSC configuration. Reproduced with permission from the American Chemical Society [16].

In addition to the n-type semiconductor photoanode (typically TiO_2_ placed on a transparent conducting oxide, TCO), a sensitizing dye, and a redox electrolyte, DSSCs require a counter electrode (CE) (Figure 1). The CE plays a pivotal part by catalyzing the reduction of the redox couple (I_3_^−^ to I^−^), collecting electrons from the external circuit to complete the electrochemical cycle. Thus, an ideal CE should exhibit extensive catalytic movement and excellent electrical conductivity to ensure competitive device performance [18,19]. Although Pt has long been the benchmark CE material, it suffers from several drawbacks, including high cost due to its low abundance and susceptibility to corrosion in iodide/triiodide electrolytes, which limit its large-scale application [20,21]. To address these limitations, extensive research has focused on developing alternative CE materials. Numerous efforts have explored carbon-based materials (e.g., carbon black, mesoporous carbon, graphene and carbon nanotubes), polymers and polymer–Pt composites and transition metal-based compounds such as sulfides, carbides, nitrides and oxides as alternative CE materials [22,23,24].

This review provides a comprehensive and up-to-date overview of both conventional and emerging CE materials for DSSCs. In addition to summarizing recent advances, it critically compares the preparation methods, characterization techniques, electrocatalytic properties, photovoltaic performance and advantages and limitations of different CE materials. This review is organized as follows: Section 2 introduces the photoelectrochemical process of DSSCs and the character of CEs. Section 3 summarizes the principal characterization techniques used to evaluate counter electrode materials. Section 4 comprehensively reviews conventional and emerging CE materials, including Pt-based materials, carbon-based materials, conductive macromolecules, transition metal complexes and their composites, together with their fabrication methods, electrocatalytic properties and photovoltaic performance. Section 5 discusses the limitations and remaining challenges for developing high-performance, low-cost CEs. Section 6 presents the main conclusions of this review. Finally, Section 7 presents this review’s future perspectives.

## 2. Principle and Working Mechanism of DSSC

DSSCs consist of a photoanode, which is a mesoporous TiO_2_ layer deposited on a TCO glass substrate; a sensitizing dye; an I^−^/I_3_^−^ redox couple; and a CE [18,19]. The way in which DSSCs operate is based on the principle of photoinduced charge transfer, a process similar to natural photosynthesis. Upon illumination, dye molecules absorb photons and become excited, with electrons being raised from their highest occupied molecular orbital (HOMO) to their lowest unoccupied molecular orbital (LUMO). These induced charge particles are then injected into the TiO_2_ photoanode’s conduction band due to the favorable energy alignment. The injected particles then move through the mesoporous TiO_2_ photoanode grid and are collected via TCO moving through external electrical pathways to generate electrical power. The nanostructured TiO_2_ provides a large active area for dye adsorption and therefore improves the light-harvesting efficiency [20]. At the same time, the oxidized dye molecules are regenerated by a redox electrolyte, generally an I^−^/I_3_^−^ pair, which donates electrons and returns dye to its neutral state before recombination can take place. The I_3_^−^ species formed in this way diffuse toward the CE, where they are reduced back to I^−^ by the electrons supplied via the external circuit. The CE, which is frequently coated with a catalytic material such as Pt, promotes this redox reaction and ensures a rapid transfer of charge [21]. The overall performance of DSSCs builds on efficient light absorption, fast charge particle insertion, effective dye reduction and high catalytic activity at the CE while at the same time reducing recombination losses amid the induced charge particles and oxidized electrolyte species. Factors such as the morphology of the TiO_2_, the properties of the dye, the composition of the electrolyte and the materials of the CE all have a critical influence on the performance and long-term stability of the device [22].

### Role and Importance of Counter Electrodes in DSSCs

Tin oxide are unable to carry out the reduction of oxidized species ($I_{3}^{-}$) owing to their inferior catalytic power and limited capacity for charge transfer [23,24,25,26]. This is with indium (ITO) and tin oxide with fluorine (FTO) why a CE exhibits a dynamic part in DSSCs, because it has to collect charge particles from the outer circuit and also catalyze the reduction of the redox pair in order to regenerate the electrolyte and maintain charge neutrality in the device. To achieve an efficient reduction of the redox shuttle, an ideal CE should have a shallow charge transmission resistance (R_CT_) and high electrical conductivity, in addition to strong electrocatalytic activity [27]. These properties have a direct impact on photovoltaic parameters, namely, the short-circuit current density (Jsc), open-circuit voltage (Voc), fill factor (FF) and power conversion efficiency (PCE) of the device.

In devices, the FF is a very important parameter because its value is largely governed by the performance of the charge-transport (CT) layer, primarily because this has an effect on the cell’s series resistance (R_s_). This series resistance consists of a number of resistive elements such as the bulk resistance of electrode materials, sheet resistance of TCO substrate and various CT resistances, including that at the TiO_2_/electrolyte boundary (R_CT_(TiO_2_)), that at the CE/electrolyte interface (R_CT_(CE)), and that at the solid–solid interfaces. Furthermore, the ionic diffusion in the electrolyte, as represented by the Warburg impedance (W), is caused by the Nernst diffusion of the I_3_^−^ species. Capacitive elements also play a significant role, consisting of the interfacial capacitance (C solid) at the TiO_2_-TCO or CE-TCO junctions and the double-layer capacitances at the TiO_2_/electrolyte (C DL(TiO_2_)) and CE/electrolyte (C DL(CE)) interfaces [28]. A high series resistance reduces the device’s performance by distorting the J–V characteristics, particularly close to Voc, and, consequently, both FF and overall PCE are reduced. Experimentally, R_s_ can be obtained from the inverse of the slope of the J–V curve near the Voc region. When the conditions are optimal and R_s_ is as low as possible, the FF attains its highest value, while greater resistive losses result in a lower maximum power output [29].

(1) $$\mathrm{FF}=FF_{0}\left[1-\left(\frac{J_{\mathrm{sc}}}{V_{\mathrm{oc}}}\right)R_{s}\right]=FF_{0}\left[1-\left(\frac{R_{S}}{R_{\mathrm{CH}}}\right)\right]$$

Here, *FF* and *FF*_0_ refer to the fill factors with and without the effect of series resistance (R_s_), respectively, and *R*_CH_ stands for the normalized series resistance (*V*oc/*J*sc). Apart from resistive losses, the catalytic performance of CE is a key factor in determining DSSC performance. The CE enables the reduction of I_3_^−^ to I^−^ in the electrolyte and, thus, promotes efficient dye regeneration. This catalytic behavior is closely linked to exchange current density (J), which is inversely proportional to R_CT_, as given by the equation R_CT_ = RT/nFJ, with gas constant R, temperature T, n (=2) electrons taking part in the reaction, and Faraday constant F [30]. Consequently, an ideal CE should possess:

- Superior catalytic activity, having a large exchange current density.
- Chemical stability against corrosion and electrolyte degradation.
- Low cost and material abundance.
- Good mechanical robustness and ease of fabrication.
- Suitable optical properties, such as high reflectivity to enhance light utilization [26,31].

The choice of CE depends on the particular application of DSSCs. For instance, building-integrated photovoltaics (BIPVs) call for transparent CEs, usually based on Pt-coated TCO substrates, whereas low-cost devices prefer carbon-based materials [32,33]. In the case of high-efficiency devices, CEs having a low sheet resistance and high catalytic activity are necessary. Even though Pt is still the most effective CE material because of its conductivity and catalytic performance, its high cost, limited availability and susceptibility to corrosion in iodide/triiodide electrolytes, which causes the formation of Pt–iodide species, prevent its large-scale commercial use [34]. As a result, a great deal of effort has been put into developing Pt-free alternative CEs that achieve high efficiency at a reduced cost. Among the promising options are carbon-based materials, polymers, metal oxides, nitrides, carbides, and transition metals [35,36,37,38].

## 3. Counter Electrode Characterization Tools

### 3.1. Structural and Physicochemical Characterization of Counter Electrodes

Before evaluating the electrochemical performance of CEs, their crystal structure, morphology, microstructure, surface topography and elemental composition should be investigated. As these physicochemical properties have a great influence on the catalytic activity, electrical conductivity, charge-transfer resistance and long-term stability of the CEs in DSSCs, various structural characterization techniques are usually employed together with electrochemical measurements in order to establish the relationship between CEs’ material properties and photovoltaic performance.

**X-ray Diffraction (XRD):** It is the most commonly used technique for investigating the crystal structure, phase purity, crystallite size and preferred crystal orientation of CE materials, especially for confirming metal sulfides, carbides, nitrides, oxides, carbon-based composites and Pt-based nanomaterials. Furthermore, changes in the intensity or width of the diffraction peaks provide useful information regarding crystallinity, phase transformation, and structural defects, all of which have a significant impact on the electrocatalytic activity of the CEs.

**Scanning Electron Microscopy (SEM):** SEM allows investigation of the surface morphology, film uniformity, porosity, thickness and microstructure of CE materials. The observations obtained by SEM help to clarify the relationship between surface morphology and catalytic performance. Because highly porous and interconnected structures usually result in a greater electrochemically active surface area and allow better electrolyte penetration, the R_CT_ is reduced and catalytic activity is improved.

**Energy-Dispersive X-ray Spectroscopy (EDX):** EDX is usually combined with SEM in order to determine the elemental composition and the spatial distribution of CE materials. Elemental mapping shows that the catalytic components, the dopants and the composite constituents are uniformly distributed, while compositional analysis proves that the heteroatoms or secondary phases which are responsible for enhancing catalytic activity and electrical conductivity have been successfully incorporated.

**Transmission Electron Microscopy (TEM)** offers high-resolution data on the size of nanoparticles, the crystal lattice structure, the lattice fringes, and the heterointerfaces present in nanostructured CEs. High-resolution TEM (HRTEM) can directly show the crystallographic planes and any structural defects, while selected-area electron diffraction (SAED) verifies the crystal phase and crystallinity. Such analyses are especially important when dealing with Pt nanoparticles, graphene composites, carbon nanotubes and transition metal nanomaterials.

**Atomic Force Microscopy (AFM)** is employed for the characterization of the surface topography and roughness of CE films at the nanometer level. It gives quantitative data on surface roughness, grain size and film homogeneity, all of which affect electrolyte wettability, catalyst loading and the effective electrochemical surface area. When the surface roughness is optimized, this usually leads to efficient electrolyte diffusion and improved catalytic performance.

After the structural characterization is performed, the electrochemical properties of the CEs are investigated by means of techniques including electrochemical impedance spectroscopy (EIS), cyclic voltammetry (CV), Tafel polarization and J–V measurements. Such techniques yield quantitative data regarding charge-transfer kinetics, catalytic activity and photovoltaic performance.

### 3.2. Electrochemical Impedance Spectroscopy

EIS is used to investigate how a system responds under the influence of an AC bias over a range of frequencies; the impedance is shown using a Nyquist plot, this plot displaying the real (Z′) and imaginary (Z″) components, or by means of a Bode plot, which shows the phase behavior. If the impedance data are adapted to a corresponding circuit, it is then possible to determine important electrochemical parameters such as series resistance (R_s_), R_CT_ and capacitance (C) [39]. These parameters provide information about interfacial charge-transfer processes, catalytic activity, and the lifetime of charge carriers in DSSCs. A high interfacial resistance leads to a reduction in both Jsc and FF, thereby lowering the overall device efficiency. Conversely, efficient CEs have low values of R_CT_ and R_s_ due to higher catalytic activity and faster redox reactions.

### 3.3. Cyclic Voltammetry (CV)

CV is used to evaluate the catalytic motion, ion diffusivity, and active surface area of CE materials in DSSCs [40]. In CV measurements, redox processes are characterized by anodic (oxidation) and cathodic (reduction) apexes detected in voltammograms. The cathodic peak corresponds to reduction (I_3_^−^ to I^−^), while the anodic peak represents the oxidation of iodide to triiodide. These reactions are respectively expressed as:

(2) $$I_{3}^{-}\;+2e^{-}\;⇌3I^{-}$$

(3) $$3I^{-}\;⇌\;I_{3}^{-}+{2e}^{-}$$

The details concerning peak current density and peak-to-peak parting refer to the catalytic efficiency and CT kinetics of CE material.

### 3.4. Tafel Polarization

The electrocatalytic activity and CT kinetics of CE materials in DSSCs can be determined by means of Tafel polarization. The diffusion coefficient of triiodide ions (D_I3−_) in electrolytes is obtainable from the diffusion-limited current density when the cell is working under symmetric cell conditions. The relationship between J_lim_ and D_I3−_ is given by Fick’s diffusion law as:

(4) $$J_{\lim}=\frac{2\mathrm{nF}\left[I_{3}^{-}\right]D_{I_{3}^{-}}}{l}$$

In the equation, n represents the number of electrons transferred, *F* stands for the Faraday constant, [$I_{3}^{-}$] is the concentration of the triiodide ion (typically 50 mM), and *l* is the distance between the photoanode and the counter electrode (approximately 40 μm). In order to allow efficient redox reactions, an ideal counter electrode should have a high J and a low R_CT_. Carbon-based materials such as carbon nanoflowers (CNFs) exhibit better catalytic performance owing to their bulky surface area and the occurrence of dense active-edge networks. These features enhance the redox kinetics and, thus, result in a higher Jsc than is attainable using conventional Pt-based CEs. The various characterization techniques, the information obtained from each of them, and the importance of this information for evaluating the properties of CEs in DSSCs are presented in Table 1.

## 4. Various Types of Counter Electrodes

In this section, the preparation methods, structural characteristics, electrocatalytic properties and photovoltaic performance of the major classes of CE materials are discussed together to provide a direct correlation between fabrication strategy and device performance.

### 4.1. Bulk Pt as the Counter Electrode

Pt has been considered one of the best CE materials exhibiting the best possible attributes, namely, stability in environmental conditions, effective optical properties, and electrical conductivity of an ideal counter electrode for highly efficient DSSCs. Examples of applications where bulk Pt is used as a catalyst are converters in vehicles, chemical reactions and operatory instrumentation, among others [46]. A thickness of 450 nm of Pt is deposited by sputtering, which results in a columnar structure with a low R_CT_ (0.05 Ω cm^2^) and high capacitance (370 (µF cm^−2^) where Li^+^ (cation) and acetonitrile (solvent) are used. Although this process generates a highly effective area, the total active area is reduced by increased porosity due to the higher Pt thickness, rather than by a thin Pt coating on TCO. Moreover, the electrolyte and charge-transfer reactions at the CE are mismatched at times according to different research groups [47,48]. It has been suggested that a 2 nm Pt thickness is sufficient to reduce R_CT_ to 2 Ω·cm^2^, compared to 25 MΩ·cm^2^ when only TCO is used as a CE [49]. So, using such a bulk Pt as the CE is not the first and foremost option, because reducing production costs is a key aspect of the mass production of DSSCs. A comprehensive study on Pt leaf, Pt plate, and sputtered Pt CEs from bulk Pt was conducted by K. Shimada et al. [50]. The Pt leaf used as the CE showed a PCE of 4.78%. The study also showed that Pt plates and sputtered Pt displayed PCEs of 4.11% and 4.40%, respectively.

### 4.2. Pt Nanoparticles

Pt nanoparticles exhibit a high active surface area, low optical absorption, low charge-transfer resistance, high electrical conductivity and excellent stability, which makes them superior CE materials compared with many other noble metals and bulk Pt. The two most important parameters for the efficiency of a DSSC are R_CT_ at the CE/electrolyte interface and the diffusivity of $I_{3}^{-}$ into the electrolyte solution [51,52]. Although cations and solvents have a significant impact on the aforementioned attributes, Pt thickness and the deposition process also contribute substantially to them. A variety of Pt deposition techniques have been investigated to achieve optimal electrical conductivity, a catalytic response toward $I_{3}^{-}$, and the high mirror-like reflectivity of Pt [15,26,37]. A comparative study on the characteristic properties of platinized TCO as a CE produced by electron-beam evaporation (2–40 nm), DC magnetron sputtering (2, 40, 450 nm) and the thermal decomposition (2–10 nm) of H_2_PtCl_6_ from isopropanol [26] indicate that coating using Pt nanoparticles between 2 and 3 nm can provide high adhesion, optimum porosity, a standard R_CT_ (≤10 Ω.cm^2^) and high transparency for DSSC application [46,49,53,54]. Different Pt fabrication techniques, including sputtering (Pt layer thickness: 50 nm, PCE of 5.17%) [53], pulse current electrochemical deposition (estimated Pt film thickness: 40 nm, PCE of 5.03%) [55], spray coating (Pt layer thickness: 50 nm, PCE of 6.17%) [56], electrochemical reduction (Pt nanourchin diameter: 100–300 nm, PCE of 9.39%) [57], thermal decomposition of (H_2_PtCl_6_) [58] (micropore diameter: 100–150 nm, PCE of 8.15%), and a bottom-up synthetic approach (PCE of 4.75%) [59], significantly influence the morphology, nanostructure, surface area and electrocatalytic properties of Pt CEs, thereby affecting DSSC performance. The thickness of the CE is an important optimization parameter because it affects electrical continuity, catalytic site density, electrolyte accessibility, optical transparency, material consumption, and charge-transfer resistance. For Pt-based CEs, thicknesses ranging from approximately 2 to 450 nm have been investigated using thermal decomposition, electron-beam evaporation and sputtering. Ultrathin Pt coatings of approximately 2–3 nm can already provide sufficient catalytic activity and low charge-transfer resistance when a continuous, well-adhered catalytic layer forms. By contrast, thicker Pt films may increase surface coverage and electrical continuity but can reduce transparency, increase Pt consumption and modify film porosity. Consequently, a universal optimum thickness has not been established. The optimum value depends on the deposition method, Pt morphology, substrate surface roughness, electrolyte composition, required transparency, and device architecture. In general, the best performance is obtained at the minimum thickness that ensures continuous electrical contact, sufficient accessible catalytic sites, low R_CT_ and good mechanical adhesion.

Studies show that these processes require a high amount of Pt resources, which restricts the potential use of the rare material Pt as a CE. Some proposed deposition methods may be considered [53] as a means to lower Pt amount requirements (100–100 µm cm^−2^), which is promising for low-cost fabrication [29,30]. These methods may either omit total use or require a small amount of Pt to produce transparent CEs compared to sputtering deposition. But the FF of the cell decreases significantly. Sputtering techniques also require a large volume of raw materials corresponding to the expected film thickness and expensive devices to achieve vacuum pressures of 10^−4^ Pa. It is worth mentioning that A. Iefanova et al. [54] proposed spray coating Pt nanoparticles onto a hot FTO substrate and annealing at 450 °C (15 min.). The sputtered Pt CE substrate exhibited no transparency at all, compared to a cumulative 80% transparency of the spray-coated Pt-ITO CE film, as supported by their corresponding UV-Vis spectra. The spray-deposited (6.17%) and sputtered (6.36%) Pt CE devices produced almost identical PCE values, whereas the former required less material consumption (>85%) than the latter.

The large surface coverage of Pt on TCO is also a very important aspect because it facilitates a more active catalytic area for triiodide reduction, thus lowering R_S_ and R_CT_—or increasing current density, in other words—which can be achieved by spray coating. The Pt NPs mimic the spherical texture of FTO without further agglomeration of the Pt NPs due to the faster evaporation of the solvent in the spray coating process [60] yielding high R_CT_ and R_S,_ which affect the total efficiency of the device. Moreover, changes in the nanomaterials’ dimensionality, as shown especially in 3D structures like nanowires [61], nanoflowers [62], nanotubes [63,64] and nanocups [65], offer interesting perspectives. The benefit of such arrangements is a higher active surface area, which is the key aspect for CE. Pt NPs on a cylindrical halloysite nanotube (HT) clay template, a naturally occurring mineral, are also a classic example of Pt-based CEs for DSCs [66]. Morphological attributes, such as a large active area, penetrability, and charge exchange, are well recognized in different fields of application. It is worth mentioning here that Nagarajan et al. fabricated Pt/HT CEs on an HT aluminosilicate template. The deposited Pt NPs on the HT followed the template to produce chain-like Pt nanostructures which were highly intersected. Devices with Pt-HT CEs exhibited a higher PCE of 9.5% than the plain Pt CEs (8.2%) using a liquid-type electrolyte.

### 4.3. Pt Composite Materials

Huge attention has been given to the catalytic performance enhancement of the Pt CE through the formation of Pt nanoparticle–C and Pt–polymer composites instead of bulk Pt. This concept was first introduced by Yen et al. [67]. Using graphene (G) as an antecedent and Pt as a structural unit, this group prepared a Pt–graphene (Pt-G) composite on a conducting oxide to form CEs. This method proposed by Chao et al. [68] is known as the “water-ethylene glycol method”. The outcome of such a composition was interesting. It was observed that the Pt-G composite exhibited improved catalytic activity due to a higher level of charge transportation properties and a higher specific active area with a lower surface contact angle compared to Pt nanoparticles and G individually. Moreover, the highest separation (∆Ep) value of the Pt-G system (80 mV) is much lower than that of Pt (159 mV) and G (110 mV), which indicates a higher degree of charge transmission and density of exchanged current unveiled from CV analysis. Meanwhile, the composite material has a decreased R_CT_ (0.67 Ω cm^2^) and a low R_d_ (0.023 Ω cm^2^). These two phenomena are responsible for the enhanced FF of the respective device, offering 6.35% efficiency compared to Pt CEs (5.47%). Carbon nanotubes (CNTs) inherently possess superior electrical conductivity and outstanding physical robustness. As an alternative form of CNTs, multiwall carbon nanotubes (MWCNTs) have demonstrated these attributes as well. This occurs due to their astonishingly high specific surface area and rapid CT characteristics [69,70]. The capping of Pt nanoclusters on conducting oxide by poly-N-vinyl-2-pyrrolidone (PVP) was introduced by Wei et al. [71]. This two-step dipping is a simple technique because the solution is easily adjustable. Devices with PVP/Pt CEs produced a PCE (4.32%) paralleling that of sputtered Pt CEs (4.55%), requiring only one-tenth of the amount. Lan et al. [72] conducted a comprehensive study among PVT/PT, Solaronix Pt-catalyst T/SP and sputtered Pt as CEs. The device with PVT/PT surpassed the PCE (5.28%) of the previous study.

**Figure 2:**
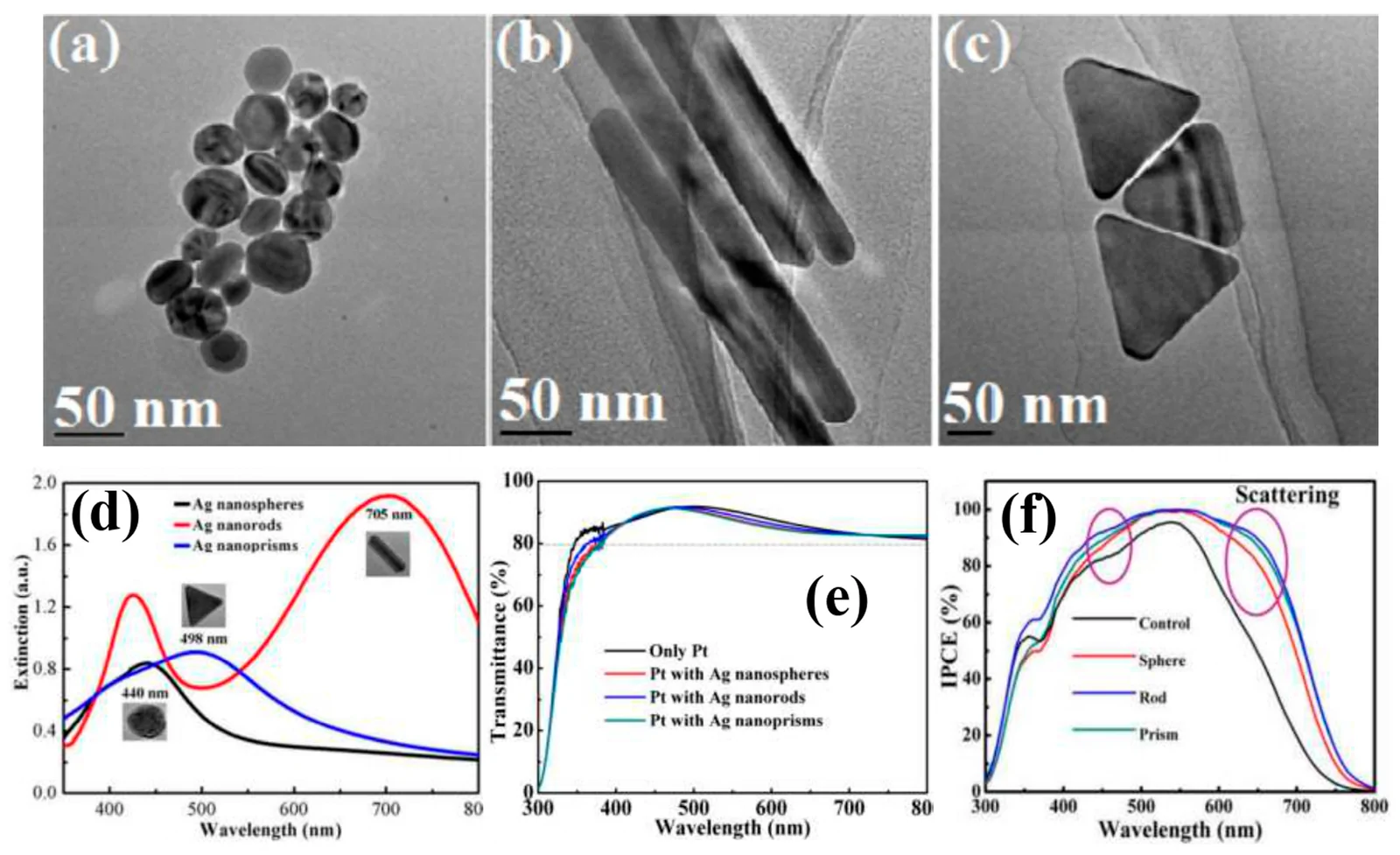
TEM images of Ag (**a**) nanospheres, (**b**) nanorods and (**c**) nanoprisms; (**d**) extinction spectra; (**e**) transmittance spectra; and (**f**) incident photon-to-current conversion efficiency (IPCE) spectra of Ag nanostructures. Reproduced with permission from the American Chemical Society [75].

The use of polymers poly(oxyethylene) (POEM) and Pt NP-MWCNT composite as CEs for DSSCs was proposed by Huang et al. in 2010 [73]. A reaction between poly(oxyethylene)diamine (POE 2000) and 4,40-oxydiphthalic anhydride (ODPA) was responsible for forming POEM. The POEM assisted in improving the electrocatalytic performance of Pt NP-MWCNT composite CE, enhancing the non-covalent bonding between the platinum nanoparticle and the MWCNTs. The efficiency of the DSSCs with Pt NP-MWCNT CEs reached over 8.00%, while the Pt–carbon black CE showed 6.72% [74]. This outcome was clearly defined by the reduction and oxidation peaks of $I_{3}^{-}$ in CV analysis. Pt was blended with different types of Ag NPs such as nanospheres, nanorods, and nanoprisms (Figure 2a–c) using the drop-casting method from precursor solution to form CEs in DSSCs by D. Ganeshan et al. [75]. The PCEs of the introduced Ag NP devices were 8.10% (nanospheres), 8.68% (nanorods) and 8.55% (nanoprisms), and those were higher compared to the devices without Ag NPs (7.60%). The device with Ag nanorods/Pt was able to display the highest efficiency by means of improved Jsc and FF due to its favorable aspect ratio along with strong disseminating properties. TEM analysis confirms an average diameter of 40 nm for Ag nanospheres (Figure 2a). In addition, Ag nanorods (Figure 2b) display a diameter and length of 20 nm and 90 nm, respectively. The edge length of Ag nanoprisms is 60 nm (Figure 2c). UV-Vis spectroscopy analysis revealed that the highest absorption peaks of the Ag nanospheres and nanoprisms were 440 and 480 nm, respectively. But, interestingly, the nanorods formed exhibited two different peaks at 425 nm and 750 nm (Figure 2d) for the transverse and longitudinal modes, respectively. The bathochromic shift due to the presence of scattering and plasmonic effects in the absorption spectra for all the Ag NPs is responsible for the better absorption profile. The transmission spectra of all three Pt/Ag NP CEs are very much similar to that of Pt CE only, showing over 80% transmittance (Figure 2e). Again, the device with Ag nanorods presented the highest PCE, around 14%, compared to that of pure Pt CEs. PCE increases of 8% and 12% were obtained in CE devices with Ag nanospheres and Ag nanoprisms, respectively. The particle dimensions, structural morphology, plasmonic effects, and scattering efficiencies of Ag NPs result in a decrease in R_sh_ and strong ohmic contact within different layers in devices, which results in an improved PCE and IPCE (Figure 2f). Pt nanourchins (PtNUs) ranging from 100 to 300 nm were produced as CEs by V. Dao et al. [57] on FTO surfaces through H_2_PtCl_6_ reduction by formic acid in ambient conditions. The PtNUs consisted of plentiful Pt NWs with a diameter of 2 nm and a length of 12 nm. It was found that the PtNU CE exhibited a very low R_CT_ for DSCs. The PCE of the fabricated device was 9.39% when using PtNUs as CEs. Then, devices with CEs of sputtered Pt exhibited a PCE of 8.51%. The (111) plan of Pt NWs on FTO as CEs is a very crucial aspect for achieving ideal photocatalytic activity. C. Chiang et al. [76] designed and prepared PtCoFe, specifically a Pt_49_Co_23_Fe_28_ NW conformation different from the other two different alloying material compositions, which consisted of higher (111) planes. The CV demonstrated a lower oxidation–reduction potential energy of 0.53 V for the aforementioned CE, certifying enhanced catalytic activity, which resulted in the highest J_sc_ of 26.21 ± 0.21 mA cm^−2^. A DSSC with Ru(II) dye PRT-22 as an absorber layer and Pt_49_Co_23_Fe_28_ as a CE produced a PCE of 12.29%, while a Pt CE generated a PCE of 10.60%. A composite of Pt and ZnO as a CE was fabricated onto FTO via a nitrogen DC-pulse atmospheric pressure plasma jet (APPJ) by Lee et al. [77]. Though a device with this CE did not significantly outperform (5.27%) the device with a pure Pt CE (5.29%), it required less heat treatment time (30 s) for functionalizing compared to the latter one (15 min). A composite of Pt NPs and Si NWs as CEs was studied by L. Yang et al. [78] in order to extend the active surface region and photocatalytic activity of Pt CEs. It showed that the presence of the Si-H bond helped the reduced metal ions to settle down on Si NWs rather than become clustered during catalysis. Electrochemical characterization indicates that the Pt/Si NW CE exhibits higher *I*^−^/$I_{3}^{-}$ activity. The Pt/Si NW CE-based DSSC showed a 3.8% higher PCE than the controlled Pt CE. The DSC with the Pt-10/PEDOT CE composite showed a PCE of 6.77%. This value of PCE for the Pt-10/PEDOT CE is higher than that of novel Pt and novel PEDOT CEs. Its lower absorption is beneficial for double-sided DSCs in building-integrated photovoltaics (BIPVs) [79]. Very recently, Shimada et al. [80] have developed a facile and low-cost single-step dipping method to prepare 3-mercaptopropyl triethoxysilane (MTS)–Pt composite CEs for DSSCs. DSSCs with MTS-1-Pt CEs showed a PCE of 8.28%, which was almost identical to that of a sputtered Pt CE (8.55%) owing to the equivalent electrochemical response toward the iodide redox couple [80].

The way in which Pt CEs adhere to the substrate and their long-term stability depend greatly on the method of fabrication. Pt films obtained by sputtering tend to have good adhesion to FTO substrates because the platinum is deposited directly on the conductive surface, which leads to good mechanical durability and stable electrocatalytic performance over long periods of DSSC operation. By contrast, Pt leaf and Pt plate electrodes are normally attached to the substrate by means of physical contact or using an adhesive, and this can result in comparatively weaker interfacial adhesion and a greater tendency to delaminate after the devices have been operated for a long time or have been exposed to repeated thermal and mechanical stress. Although direct comparative studies carried out under the same DSSC operating conditions are still limited, stronger interfacial adhesion is generally associated with better durability and more stable photovoltaic performance. The catalytic activity of Pt-based CEs is greatly influenced by their composition, morphology, and the method of fabrication. Conventional bulk Pt has very good intrinsic catalytic activity and electrical conductivity but is limited by its relatively small specific surface area. On the other hand, Pt nanoparticles provide a larger electrochemically active surface area, a lower R_CT_, and a greater number of catalytic active sites, thus improving the kinetics of triiodide reduction. Furthermore, Pt composite CEs benefit from the synergistic effects between Pt and the conductive or porous supporting materials, which leads to better electron transport, greater electrolyte accessibility, more effective catalyst utilization, less Pt loading, and excellent long-term stability. Consequently, Pt nanoparticles and, in particular, Pt composite CEs generally exhibit better electrocatalytic performance than conventional bulk Pt.

### 4.4. Carbon Materials

The multiple forms of carbon, such as diamond, graphite, and noncrystalline carbon, are well recognized for their use for many different purposes. In the search for low-cost and earth-abundant materials to replace the Pt noble component as the CE in DSCs, carbon is standing at the top of the list. A few of the very attractive attributes of carbon, such as high abundance (sixth) in the Earth’s crust, outstanding low cost, nontoxicity for the environment, and non-corrosiveness, have been well recognized in CEs for DSCs for a long time. Moreover, its response to the catalytic conversion of the electrolyte is brilliant. All the different variations of the carbon family have shown competitive photovoltaic performance while being used as CEs in DSSCs [81]. By using CEs made of carbon black dispersed into graphite powder, a PCE of 6.7% in a series connection was achieved by Gratzel et al. [82]. The high porosity of such materials is packed with an electrolyte solution to enhance catalytic activity via high surface area and roughness. A comparative study of graphite and activated and glassy carbon (1000–2000 m^2^ surface area) as CEs was performed by Imoto et al. [83]. The activated carbon had a threefold higher active area compared to the other two versions of carbon. The addition of carbon into that CE demonstrated superior J_sc_ and FF.

#### 4.4.1. Carbon Black

Carbon black (CB) is used predominantly as a filler to reinforce tires and in other rubber products. It is also used in plastic, paint and toner for printers [84]. It is paracrystalline in nature, with a high surface-area-to-volume ratio and excellent electrical conductivity. Moreover, the electrocatalytic reduction of the I^−^/I_3_^−^ redox couple primarily occurs at the edges of the carbon black (active sites) [85]. These edge sites possess a high density of unsaturated carbon atoms, structural defects, dangling bonds, and oxygen-containing functional groups, which provide favorable adsorption sites for I_3_^−^ ions and facilitate rapid electron transfer during the redox reaction. By contrast, the basal planes are relatively inert because of their highly ordered graphitic structure. Thus, CE thickness plays a major role in catalysis, as increasing the thickness increases the number of accessible active sites, thereby reducing the R_CT_ and improving the J_SC_ and FF. Wu et al. [77] proposed a composite of Pt and CB as a CE for DSSCs on bare FTO glass deposited by spin coating, which requires ethyl cellulose (EC) to control Pt/CB viscosity. The 1:3 ratio of Pt: CB was proven to be effective. But ethyl cellulose (EC) showed a great impact on device performance. The ideal elemental ratio of 30:4 for the (Pt/CB):EC CE yielded a PCE (8.06%) almost similar to that of a conventional Pt CE (sputtered, 8.17%) by a simple spin coating process. Rahman et al. [86] introduced Pd NPs (NCB@Pd) as CEs, which were surrounded by nitrogen-doped CB (NCB). The CEs of CB, NCB and NCB@Pd were created on FTO glass in a sponge-like matrix. The device showed a PCE of 7.95% that was higher than that of the Pt CE (7.85%). It was observed that the NCB@Pd CE had superior catalytic activity compared to the Pt CE and eased the *I*^−^/$I_{3}^{-}$ redox reaction quickly. Furthermore, the enhanced surface engineering of NCB@Pd was responsible for lowering the charge recombination and CT through the improvement of the Pd NP properties [87]. Meanwhile, the CE substrate is very important. Nowadays, most of the fabrication that is performed on TCOs such as FTO or ITO is costly and non-flexible, which restricts the deployment of their full potential. Though metal substrates can be tuned and used, the corrosive nature of the iodide redox couple may degrade them. So, carbon could be a suitable alternative substrate that has anti-corrosive attributes and low sheet resistance. Stainless steel substrates SUS-316 and SUS-304 were coated with carbon, and they showed PCEs of 9.15% and 8.86%, respectively, as a CE DSC device. Jaafar et al. [88] recently proposed a composite of CB-TiO_2_ as a CE for DSCs. As an alternative to Pt CEs, the device with CB-TiO_2_ CEs exhibited J_SC_ = 6.10 mA/cm^2^, V_OC_ = 0.51 V, FF = 0.89 and η = 2.77%, which is higher than that observed with the device with Pt CEs (1.42%–2.00%) [89,90]. It is observed that temperature impacts the adhesion between CB-TiO_2_ and the substrate. A 15 wt% CB-TiO_2_ composite annealed at 525 °C might exhibit superior catalytic activity due to enhanced porosity. The reduced RCT is accountable for the improved J_SC_. An acetylene CB–graphite-based CE was studied by Don et al. [91]. The insertion of CB particles into graphite cement improved the conductivity of the acetylene CB–graphite composite. The interconnected graphite flakes are responsible for the enhanced $I_{3}^{-}$ reduction. AB/CB particles also offer a large surface area to provide high electrochemical performance in DSSCs. Overall, the highest PCE of 5.06% was achieved with a CE of acetylene CB–graphite (3:1), which was equivalent to that observed with the device with a Pt CE. These findings suggest that acetylene CB–graphite composite is a promising CE for low-cost DSSCs.

#### 4.4.2. Mesoporous Carbon

**Figure 3:**
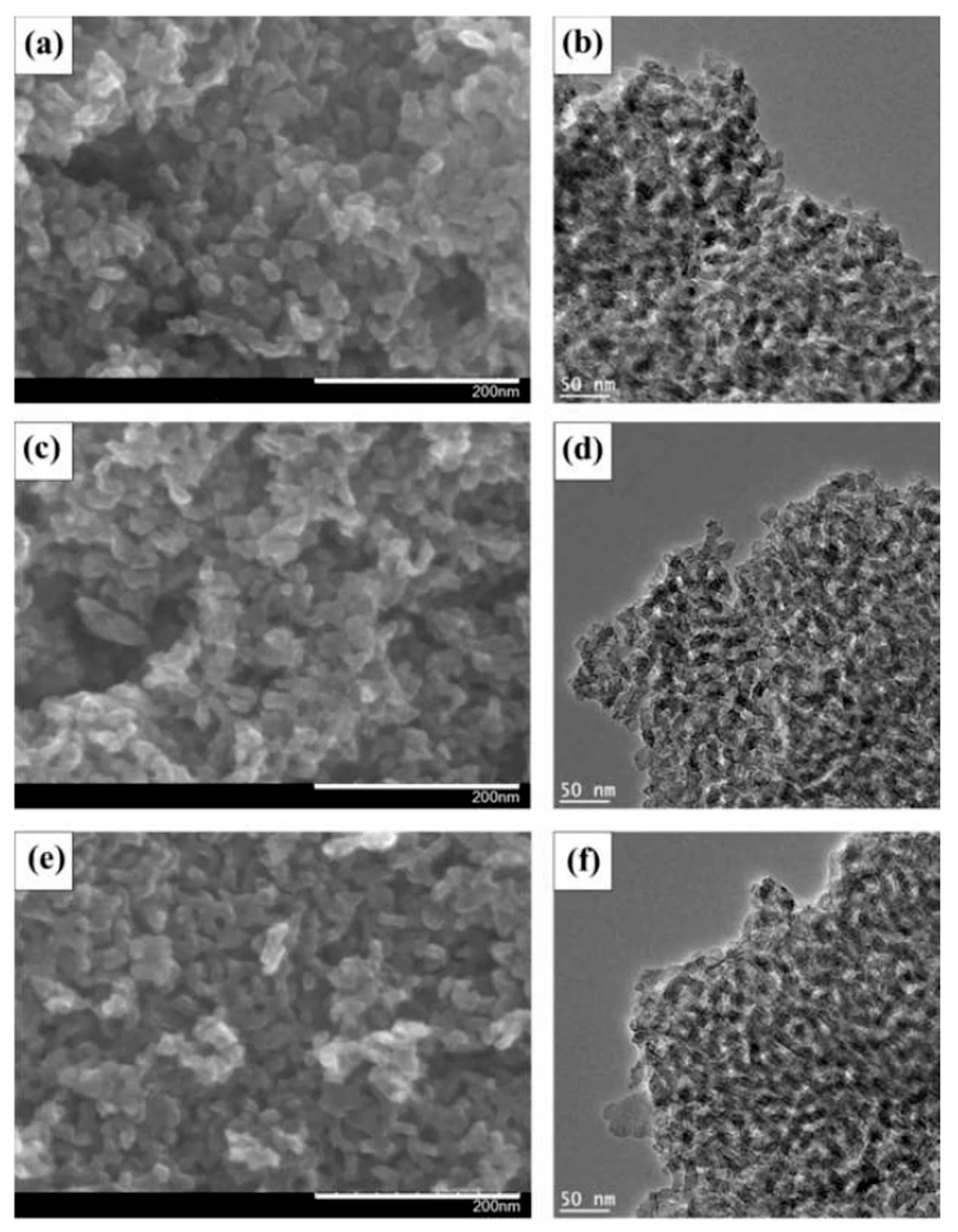
FE-SEM images of TeMC composites carbonized at (**a**) 700 °C, (**c**) 800 °C and (**e**) 900 °C, together with the corresponding HR-TEM images shown in (**b**), (**d**) and (**f**), respectively. Reproduced with permission from Ref. [95]. Copyright 2020 MDPI.

Mesoporous carbon has gained huge attention compared to other carbon elements. This variety of carbon contains a bulky cumulative surface area and a porosity that can be turned into a different pore radius. Mesoporous carbon is generally produced by nanocasting, while a template is used to obtain such a carbon form. This synthesis route produces consistent, highly porous and interconnected apertures. The electrolyte penetration is due to the large area in CEs, which results in more efficiency (7.1%) compared to the activated carbon CEs [92]. Deploying carbon with different morphologies can decrease the charge of the CE and advance the performance of the DSC device [93]. Recently, Wu et al. [94] suggested the doping of MC with oxygen and sulfur, which introduced defects in morphological symmetry with more disordered carbon. As a consequence, the electrolytic active area is increased, which allows faster redox reaction to take place. Moreover, the addition of sulfur heteroatoms to this structure could provide ample electroactive area and also reduce the oxygen-bound carbon pillar for a smooth reduction of the redox couple. DSCs with dual oxygen- and sulfur-doped carbon CEs exhibited a PCE of 10.2%, higher than that of a Pt CE (9.4%). A transparent CE using MC doped with Te (TeMC) was prepared and applied in DSCs by Kim et al. [95]. The pyrolysis of the polyacrylonitrile-block-poly(n-butyl acrylate) (PAN-b-PBA) block copolymer in the Te metalloid produced TeMC in a nitrogen atmosphere. The CE for bifacial DSCs was electrocatalytically active, with a low R_CT_ for transferring electrons from the external circuit to the CE surface; highly transparent to enhance back-side radiance; and electrochemically stable for large-scale production. The TeMC CEs of optimized thickness and treatment under altered carbonization temperatures showed excellent photoelectrical and photophysical properties, with brilliant photovoltaic performance as bifacial DSSCs. Figure 3 shows FE-SEM (a,c,e) and HR-TEM (b,d,f) images of the TeMC materials with different carbonization temperatures. The TeMC films possess an interconnected porous structure in 3D and homeomorphic assemblies. These structures are more clearly identifiable in the TEM images (Figure 3b,d,f), having a constant pore size that is uniformly distributed in the MC framework. The TeMC treated at 900 °C has a higher specific area and micropore (526.79 m^2^ g^−1^ and 0.87 cm^3^ g^−1^) capacity than TeMC carbonized at 700 °C (399.68 m^2^ g^−1^ and 0.80 cm^3^ g^−1^) and 800 °C (455.05 m^2^ g^−1^ and 0.78 cm^3^ g^−1^). The surface of the carbon framework quickly disintegrated, increasing the specific surface area at high temperatures. The R_CT_ value from EIS suggests that the TeMC CE has a similar resistance compared to the Pt CE. This proves its better electrochemical constancy. DSSCs with TeMC CEs exhibited better efficiency (9.43%) compared to DSSCs with Pt CEs (9.23%) due to superior electrochemical characteristics.

#### 4.4.3. Graphene

A hexagonal arrangement in the honeycomb structure of SP^2^-hybridized carbon atoms forms a 2D sheet of graphene. Graphene has become the center of research across different fields since its discovery in 2004 [96]. Graphene possesses extraordinary physical and electrical properties such as high charge carrier mobility (~10^4^/Vs) [96,97], a high specific surface area (2630 m^2^/g) [98], exceptional thermal conductivity (3000 W/m K) [99], a high Young’s modulus (~1000 GPa) [100], and very low optical absorbance (2.3%) [101]. All these attributes are highly expected from an alternative Pt CE for DSSCs. In particular, the high conductivity and surface area are responsible for reducing R_CT,_ which results in quick CT to the respective layers. There are many ways to synthesize identical graphene nanosheets (GNs) for different uses as CEs for DSSCs. Zhang et al. [102] first studied the adaptability of GNs as CEs for DSSCs. GNs were synthesized via complete separation from graphite oxide layers, followed by reduction in the presence of hydrazine. The Raman spectra reveal the graphitic topographies of GNs, which are disordered and provide a higher specific surface area. The GN CE was prepared by screen printing on FTO glass and annealing at 400 °C, which exhibited an R_CT_ of 1.2 Ω and a PCE of 6.8%. Annealing temperature has a significant influence on the adhesion, microstructure and electrocatalytic performance of graphene CEs. During thermal treatment, residual solvents, oxygen-containing functional groups and organic binders are gradually removed, which improves the interfacial contact between the graphene film and the FTO substrate and thereby enhances film adhesion. Simultaneously, partial reduction of graphene oxide restores the sp^2^-conjugated carbon network, improving electrical conductivity and facilitating electron transport. The evolution of gaseous species during annealing also promotes the formation of interconnected three-dimensional porous graphene networks, which increase the electrochemically active surface area and facilitate electrolyte penetration. Consequently, the R_CT_ decreases, which leads to enhanced catalytic activity toward the I_3_^−^/I_3_^−^ redox reaction. However, excessively high temperatures may induce graphene sheet aggregation or degrade the porous architecture, resulting in reduced catalytic performance. Organic binders may also decompose or burn off at excessively high temperatures (400 °C), which can weaken the adhesion of graphene sheets to the FTO substrate and cause partial film delamination. There are other ways to fabricate GNs, such as microwave-assisted chemical reduction of GO [103], electrophoretic deposition (EPD) [104], etc.

#### 4.4.4. Graphene–Metal Composite

**Figure 4:**
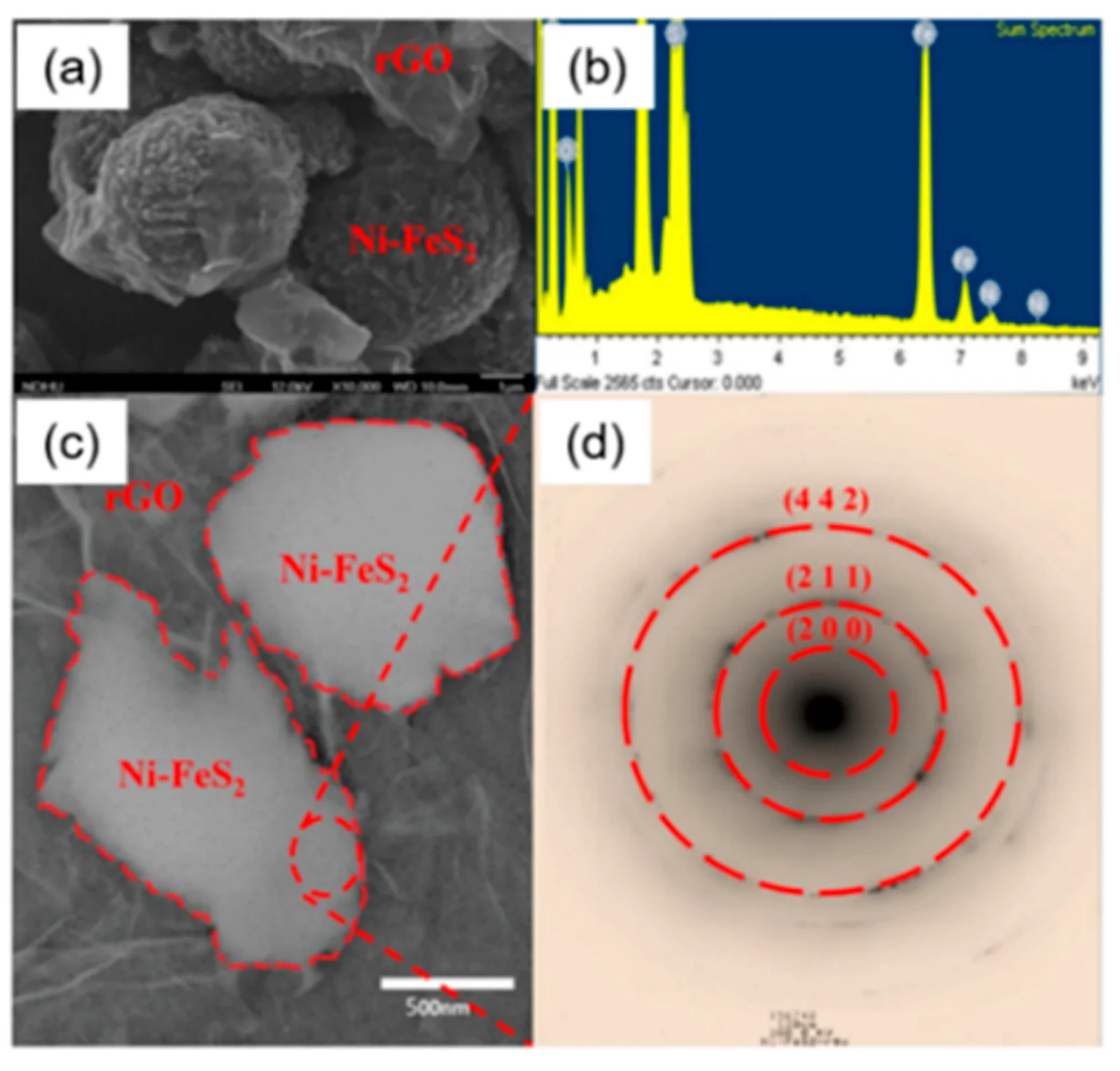
(**a**) FE-SEM image, (**b**) EDS elemental mapping, (**c**) TEM image and (**d**) selected-area electron diffraction (SAED) pattern of the Ni–FeS_2_/rGO composite. Reproduced with permission from Ref. [116]. Copyright 2020 Royal Society of Chemistry.

In order to reduce the Pt amount [105], transition metals like Ni and Co could be an alternative in conjunction with graphene to form a composite CE. The G–metal composite as a CE is advantageous for an increased CT rate, as an expected response to the reduction of the redox couple through electrocatalytic activity. Last but not least, the metal lies between graphene sheets, facilitating the diffusion of electrolytes through the G bridge/backbone and improving the close contact between the CE and electrolyte [106,107]. Graphene nanosheets (Gns) were grafted with CoS through a hydrothermal method by Rahman et al. [108]. The crafted CoS@Gns composite CE had a PCE of 7.28%, which was equivalent to the PCE of a Pt CE (7.28%). The PCE was higher than that for the CoS-only CE under the same conditions. This outcome could be credited to the magnetic properties of CoS. Moreover, grafted CoS on G performs like a backbone between layers of G, which provides mechanical support and increased triiodide reduction active sites and conductivity, resulting in a stable and high PCE. Two-dimensional reduced graphene oxide (rGO) is an outcome of the exfoliation of graphite in a hexagonal form due to sp^2^ hybridization of carbon particles [96,109]. rGO is greatly recognized for its high charge carrier transport, lower hydrophilicity and facile preparation for different applications, such as in catalyst, energy storage and conversion devices [110,111], especially as a CE [112,113]. The major drawbacks of rGO are the reduction of the specific active area and the presence of π–π stacking that enhances the rGO layer aggregation, leading to reduced catalytic active spots toward the redox couple when considered as a CE individually [114,115]. Strategies have been considered to overcome these drawbacks because this is one of the cheapest and most available materials on Earth. If those issues could be eliminated successfully, then it would be a breakthrough which could replace the more costly Pt. Some composites are Ni-doped FeS_2_/rGO (Ni-FeS_2_/rGO) [116], S-rGO/MoS_2_ [117], Ag-doped rGO (Ag-rGO) [118], and MoFe alloy-decorated rGO [119]. The proposed Ni-FeS_2_/rGO by Kumar et al. [116] was synthesized using a hydrothermal method. Figure 4a shows that the Ni-FeS_2_ nanostructures are evenly distributed and scaffolded by rGO, which is advantageous for charge transfer on the Ni-FeS_2_/rGO interface. Figure 4b shows the EDS spectrum of the NFG2 composite. The TEM image of the NFG-2 composite shown in Figure 4c depicts the Ni-FeS_2_ wrapped up in transparent rGO sheets. The SAED pattern in Figure 4d reveals (2 0 0), (2 1 1) and (4 4 2) planes observed with d-spacings of 2.77, 2.24 and 0.90 Å, respectively. The optimum composite revealed high catalytic activity with high Jsc, a limiting current, and a PCE of 7.60% for DSSCs, surpassing a Pt CE (6.69%), which resulted from lower R_CT_ resistance and a higher carrier lifetime. In another example, Dou et al. prepared G-Ni1_2_P_5_ composite CEs hydrothermally, which exhibited a PCE of 5.7% [120]. Recently, an iron carbide (Fe_3_C) wrapped with graphene (Fe_3_C@G) composite was proposed by Xu et al. [121] as a CE for DSSCs. Fe_3_C seems to have Pt-like electrocatalytic activity, high robustness, and low R_CT_ [122,123]. The group evaluated the magnetic field-induced (Fe_3_C@G-M) method for preparing the CE. The Fe_3_C@G-M composite CE yielded a PCE of 7.20%, which was competitive with that of the Pt CE (7.35%). The composite might offer uniform dispersion, providing a large electrocatalytic active area and strong adhesion between Fe_3_C. G backed up by a low R_CT_ at the hole conductor–CE edge resulted in a competitive FF. The Fe_3_C@G-M combination is a prospective low-cost CE material for replacing the more expensive Pt.

#### 4.4.5. Graphene and Polymer Amalgamated Material

Stankovich et al. [99] first reported on the graphene–conducting polymer composite as a CE for DSSCs in 2006. In this arrangement, graphene offers a reduction of triiodide through its catalytic activity, while the conductive polymer part provides conductivity. However, graphene can also be arranged as a support for polymer once dispersed. Polyaniline (PANI) and poly(3,4-ethylenedioxythiophene) (PEDOT) are the most commonly used in graphene–polymer matrix CEs. A composite of graphene and PANI was studied by Wang et al. [124], offering a PCE of 6.09%, whereas the bulk Pt CE showed a PCE of 6.88%. Recently, Bayram et al. [125] prepared graphene–PANI CEs on a single- (G 1) and multi-layer (G 5) graphene film by plasma polymerization. A transmittance of the graphene layers ranging from 97.6 to 86.6% (at 550 nm) was observed. The G 1/PANI and G 5/PANI CEs showed PCEs of 1.36% and 0.56%, respectively, which reflects that the graphene layer number had an impact on device performance. The Gr1/PANI CE displayed a better PCE than the Pt CE, as reported in [126]. Similarly, Lee et al. [127] proposed a PEDOT–graphene composite as a CE, which exhibited a PCE of 6.26% resulting from high Gr stacking to PEDOT, which promoted higher CT and catalytic activity. Again, PEDOT-PSS (3,4-ethylenedioxythiophene) poly(styrenesulfonate) is another conducting polymer where G is easily dispersed. The strong immobile attraction between functionalized G and PEDOT is responsible for such dispersion of G into the PEDOT:PSS matrix [128]. A PCE of 4.5% was achieved with G/PEDOT:PSS as a CE, while bulk Pt exhibited 6.3% in the same condition. The Co(bpy)^2+/3+^ redox shuttle offers good photovoltaic performance in DSSCs. But Pt seems to be non-optimal, and Gr alone is unable to provide the expected CE robustness. So, Kim et al. [129] infused Gr into a PEDOT PSS matrix, which helped in delocalizing electrons in the polymer framework and rapidly transferred charges. The DSSC with PEDOT: PSS-G CEs showed a higher PCE of 8.33% than the Pt CE (7.99%) under the same optimized condition.

#### 4.4.6. Graphene–Carbon Composite

Graphene, which is a strong contender for a CE, could offer great carrier mobility (hole), a non-responsiveness toward moisture and ambient oxygen, unlike Pt. Choi et al. [36] proposed a graphene–MWCNT composite as a CE, where the graphene layer was covered by MWCNT through chemical vapor deposition (CVD). The framework CE exhibited a PCE of 3%, which was higher than that of an MWCNT CE, but it involved a complex production method and potentially high manufacturing cost in the CE preparation. An easy method was conceptually used to prepare an MWNT–Gns composite CE by Battumur et al. [130]. Comparative CV studies advised that MWCNTs, graphene, and MWCT–Gns had identical oxidation–reduction peaks. A composition of 60% MWCNTs and 40% graphene exhibited a PCE of 4%. Velten et al. [131] investigated an MWCNT–Gr complex as a CE that displayed a high PCE of 8.82%, representing that an optimized ratio of MWCNT and G could significantly enhance the redox reaction by means of electrical conductivity. Meanwhile, a fiber-type CNT (CNTY) had also been studied by Kim et al. [132]. The CNTY as a CE in solid-state DSSCs produced a PCE of ~4% after optimization, which was superior in terms of PCE and stability to a Pt CE. The device architecture was Ti wire/TiO_2_/N719/electrolyte/Pt or CNTY to evaluate photovoltaic performance. The J-V curves under AM 1.5 illumination were matched consecutively. The DSSCs with CNTY as CEs showed a PCE of 4.0% (Jsc of 8.32 mA/cm^2^; Voc of 0.67 V; FF of 71.97%). By contrast, DSSC with Pt as CEs had a PCE of 2.64% (Jsc of 6.06 mA/cm^2^; Voc of 0.64 V; FF of 67.98%). DSSCs with fiber CEs displayed better Jsc resulting from expected catalytic activity toward redox phenomena and strong interaction spots, which reduced R_CT_ at the CNTY-to-electrolyte interface. The better mechanical properties and performance stability after repeated bending and high output voltage for series connection reveal that CNTY fiber-shaped DSSCs may find applications as flexible power sources for inflexible/wearable fiber electronics and energy textiles.

### 4.5. Polymer Materials

The continuation of a single bond and a double bond concurrently is referred to as conjugation, which is responsible for charge transport in conducting polymers. The conjugated bonding in a polymeric backbone facilitates conductivity. In addition, the electrocatalytic activity of such polymers to reduce $I_{3}^{-}$ mimics Pt CEs, which depends on the degree of polymerization and doping states [133,134]. Among conductive polymers, PANI, polypyrrole (PPy), PEDOT:PSS, and polythiophene (PTh) and their derivatives are widely recognized as CEs, and their performance can be found in previous reviews [135]. The inherent electrocatalytic response of such conducting polymers is largely governed by the architecture of individual monomers. As an example, PProDOT and PEDOT possess a similar monomeric structure, which is clearly different from the monomeric structure of PPY and PANI. Due to the formation of cross-linked bonding of monomers during polymerization, conductive polymers form a 3D porous framework, which is advantageous for film formation and satisfies the requirement of a very thin layer for effective electrocatalytic activity. Moreover, the morphology and structure of conductive polymers can easily be tuned by controlling preparation conditions [136]. Here, we address some recent work/investigations with these polymeric compounds, their derivatives and composites with different materials. Wu et al. [137] created a series G/PANI composites where PANI was doped with metal ions such as Mn^2+^, Co^2+^, Ni^2+^and Cu^2+^. Among the surface topologies designed—G/PANI-Mn^2+^, G/PANI-Co^2+^, G/PANI-Ni^2+^, G/PANI-Cu^2+^, and G/PANI—G/PANI-Mn^2+^ has the smallest and most uniform particle size, with a smoother surface and a higher active surface area compared to the others. Table 2 presents the photovoltaic parameters of DSSCs with these CEs. Among all the CEs, G/PANI-Mn^2+^ as a CE showed the highest efficiency, with a promising outcome.

### 4.6. Alternative Material/Transition Metal Carbides/Nitrides/Oxides/Sulfides/Tellurides

The electrical conductivity of CE materials is typically in the order of metals and alloys > nitrides > carbonaceous materials > compounds of multiple metals > conductive polymers, selenides > sulfides > oxides. Because higher conductivity results in reduced energy loss during CT, CE efficiency does not have a direct relationship with electrical conductivity. This limitation means that each individual material can be examined for use in a redox reaction or for electrical conductivity when preparing a CE. In the section that follows, we look at how the various types of materials have been improved in terms of electrocatalytic activity. Apart from carbon and its allotropes, a great number of alternative CEs to Pt have been investigated. These include CoS, NiS, MoS, NiN, MoP, Ni5P_4_, transition metal carbides (TMCs), nitrides (TMNs), oxides (TMOs), sulfides (TMSs), etc. [138,139,140]. These materials can be prepared using low-cost processing. In 1974, Levy et al. [141] found that tungsten carbide (WC) had an electronic structure that could mimic the catalytic activity of Pt. Various devices in the field of energy, such as those involved in hydro-desulfurization and the oxidation of hydrogen and methanol, have been considered for replacing Pt with transition metals. Following the use of CoS and tin in 2009, the application of transition metals accelerated [142,143]. We give an outline of the rise of various TM compounds such as carbides, oxides, nitrides, borides, phosphides, selenides and tellurides as alternative CEs in DSCs.

#### 4.6.1. Carbides

**Figure 5:**
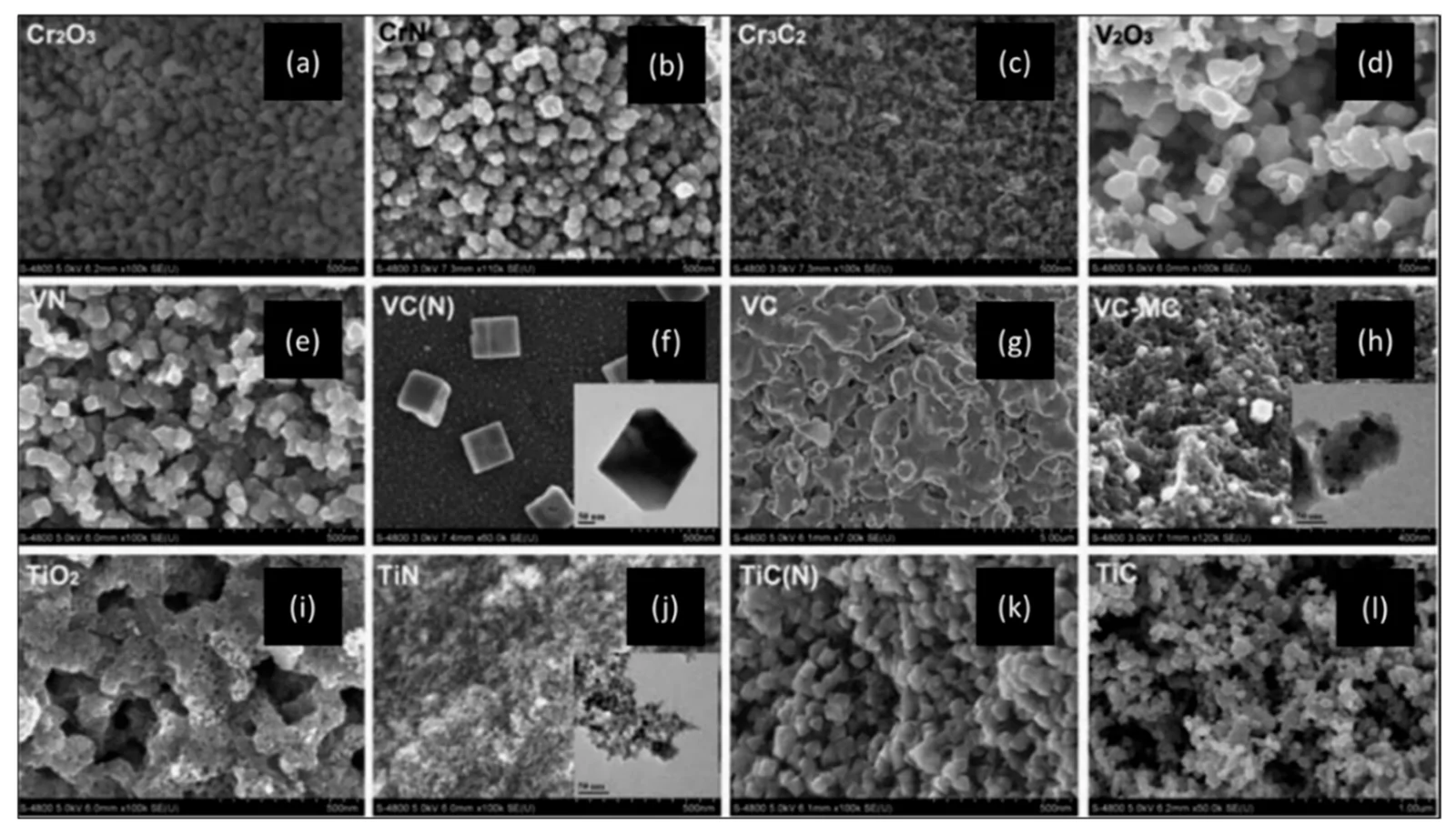
SEM images of synthesized (**a**) Cr_2_O_3_, (**b**) CrN, (**c**) Cr_3_C_2_, (**d**) V_2_O_3_, (**e**) VN, (**f**) VC(N), (**g**) VC, (**h**) VC–MC, (**i**) TiO_2_, (**j**) TiN, (**k**) TiC(N) and (**l**) TiC materials. Reproduced with permission from Ref. [138]. Copyright 2012 American Chemical Society.

WC was first used as a CE in DSCs by Lee et al. [144] in 2010. The device produced a PCE of 7.01%, while a device with a Pt CE showed a PCE of 8.23%. This reflects that WC possesses lower catalytic activity than Pt. Commercially available WC and Mo_2_C were also proposed as CEs for DSCs, and they gave PCEs of 5.35% (WC) and 5.70% (Mo_2_C), lower than that of a Pt CE (7.89%) [145]. The larger particles and lower active area might be responsible for such poor PCE. In an attempt to enhance catalytic activity sites by obtaining nanoscale carbides via using metal chloride, urea was the source of metal and carbon [138]. The synthesized Cr_3_C_2_ and M_O2_C were highly pure. But nitrogen was found in TiC, VC, and NbC, and it could be removed by increasing the sintering temperature and time [146]. The achieved PCEs with those CEs were 5.79% (Cr_3_C_2_), 5.83% (Mo_2_C), 6.52% (TiC), 6.38% (VC), 3.85% (ZrC), and 2.46% (NbC). Figure 5 display the WC and W_2_C synthesized following the above route, which resulted in PCEs of 6.23% and 6.68%, respectively [147]. Moreover, Ta_4_C_3_ was used as a CE in DSCs, and it exhibited high redox reaction activity [148]. It was observed that nanoscale transition carbide influenced catalytic activity better than the larger particle. A high PCE of 7.63% was obtained when VC was mixed with MC to form a VC–MC composite material. A combination of VC and MC could improve catalytic activity considerably. The carbides were more robust toward the new organic redox couple of di-5-(1-methyltetrazole)disulfide 5-mercapto-1-methyltetrazole N-tetramethylammonium salt T2/T^−^ compared to what was observed with a Pt CE [138]. All these materials can be considered as potential CEs in DSCs.

#### 4.6.2. Nitrides

In 2009, Gao et al. [143] prepared TiN to be used as a CE in DSSCs. The TiN nanotubes were prepared by electroplating and nitridation on a Ti sheet. The DSCs with the TiN CE produced a PCE of 7.73%, which is higher than that of the Pt CE (7.45%). In addition, a NiN CE for a DSC showed a lower PCE of 5.68% [149], which was attributed to the rigid formation of NiN nanoparticles resulting from the lower exposed catalytic surface area. While a NiN mesoporous layer was used as CE, the PCE significantly improved to 8.31%, which confirms the impact of layer porosity. The nitrides of Mo, W, and Fe_2_ were also synthesized as CEs, and the DSCs exhibited PCEs of 5.57% (Mo_2_N), 3.67% (W_2_N), and 2.65% (Fe_2_N) [140]. A mesoporous Mo_2_N nanorod CE was deposited on Ti foil by Song et al. [150]. The DSC exhibited a PCE of 7.29%, larger than that of MoN (6.48%) due to improved diffusion kinetics. Additionally, W_2_N and Mo_2_N flexible CEs were prepared on Ti foil by magnetron sputtering. The DSCs gave PCEs of 5.81% (Mo_2_N) and 6.38% (W_2_N). EIS results revealed that the Rs of W_2_N and Mo_2_N CE were only 1.8 and 1.1, respectively, resulting from Ti foil’s high conductivity and the strong grip between the Mo_2_N or W_2_N film and the Ti foil substrate [151]. Meanwhile, thermal-decomposition-processed cubic-structured γ-Mo2N, used as a CE, showed 5.36% efficiency, comparable to that of Pt (6.4%) [152]. VN CEs of different particle sizes were produced by Wu et al. [153]. The catalytic activity of the series was VN peas > VN cube > large VN for the iodide redox shuttle. The equivalent PCEs of 7.29% (VN peas), 6.53% (VN cubes), and 5.59% (large VN) were obtained. The dissimilar catalytic performance of VN materials was attributed to the altered specific surface area and the different electronic configurations, which influenced particle size. Prasad et al. [154] synthesized three-dimensional rhombus-shaped Ni_3_N nanomaterials. The DSC with a Ni_3_N CE showed a noteworthy PCE of 2.88%, as well as extraordinary device robustness. The performance of Ni_3_N resulted from good charge transport and a high number of electrochemically active surface sites for catalytic activity. These effects prove that Ni_3_N is promising for efficient CEs in DSSCs.

#### 4.6.3. Sulfides

As a flexible CE, CoS onto ITO/PEN was fabricated by Wu et al. [142]. The CoS CE presented a PCE of 6.5%, which was the same as that in devices based on Pt CEs. Moreover, CoS showed high stability in severe environmental conditions. A nanosheet (NS) of Co_3_S_4_ with a stratified shape was synthesized on FTO via a single-pot hydrothermal process in order to be used as a CE in a DSC by Wu et al. [155]. The device with Co_3_S_4_ as the CE had a PCE of 7.19%. The authors credited the laminar shape of Co_3_S_4_ NS with the improved response in charge and mass transport. The same group produced a single-dispersed nanosheet (NS) of CoS_2_ tuned into a nanoink for use as a CE in a DSSC. They reported a PCE of 6.78% and 6.40% with rigid and flexible substrates, respectively. NaBH_4_-and H_2_SO_4_-treated CoS thin film, which was prepared through electrophoretic coating and ion exchange deposition route, showed a PCE of 7.72% [156]. The exposure of CoS to a mild reducing agent and acid offered more porosity with a hexagonal prismatic structure. Thus, to facilitate a redox reaction, it increases the active surface area in many folds. The CoS CE prepared by microwave-assisted chemical bath at 100–120 °C resulted in a PCE of 8.39% compared to 7.88% for the Pt CE [157]. Laminar sulfides of Mo and W showed an encouraging performance as CEs. The DSSCs with MoS_2_ and WS_2_ CEs had a high PCE of 7.59% and 7.73%, respectively. Those values are competitive relative to a Pt CE (7.64%) [158]. Hussain et al. [159] obtained PCEs of 8.21% and 7.12% for CuS/WS_2_ and CuS/MoS_2_ composites, respectively. These values are competitive relative to Pt CE (8.74%) and the original WS_2_ (6.0%), MoS_2_ (6.3%) and CuS (6.4%). A MoS_2_/CNFs complex was synthesized using a combination of electrostatic fiber spinning and a hydrological method by Li et al. [160]. The composite resulted in packed flower-shaped morphologies of MoS_2_/CNFs. The DSSC had a PCE of 8.46%, higher than for the original Pt CE (7.65%). The authors concluded that the improved PCE originated from the enhanced charge transfer with MoS_2,_ which was validated by diffusion-limited current density through Tafel polarization curves. Thus, MoS_2_/CNFs CEs are a promising cost-effective CE alternative for future DSSCs. A MoS_2_@SnS_2_ heterostructure as a CE showed a PCE of 7.63% [161]. MoS_2_ acts as an efficient catalyst, providing synergistic properties with the active SnS_2_ NPs along with charge collection characteristics and abundant grain-edge active spots. MoS_2_ film was deposited via CVD on FTO glass substrate for DSSCs as a CE [162]. The high reflectivity of the MoS_2_ CE yielded a PCE of 7.5% (Pt CE 7.28%). A solution-processed MoS_2_ CE on a graphite paper (GP) substrate was prepared by Niu et al. [163]. The DSSC with the MoS_2_/GP CE exhibited a PCE of 6.48%, which was higher than that of the device with a pristine Pt/FTO CE (6.22%). Furthermore, MoS_2_/GP offered elevated stability due to the relatively higher crystalline structure and anchoring on the substrate. Hansol et al. [164], meanwhile, reported an efficient MoS_2_ CE prepared at 70 °C followed by near-infrared laser sintering. The DSC device with a high-temperature-sintered MoS_2_ CE showed a PCE of 5.96%, but a laser-sintered MoS_2_ CE produced an efficiency of 7.19%. Recently, hydrothermally produced MoS_2_ coupled with rGO, which was realized by Vijaya et al. [165] as a CE, displayed a PCE of 8.1%. In an attempt to fabricate a flexible DSSC, Gurulakshmi et al. proposed a CE of electrodeposited MoS_2_ assisted with ionic liquid 1-butyl, 3-vinyl imidazoliumiodide (BVImI), which exhibited an encouraging PCE of 4.84% [166]. Vanadium sulfide (VS_2_) hydrothermally synthesized by Liu et al. [167] was used as an alternative CE for a DSC. The device with VS_2_ showed a PCE of 6.24% (Pt 6.44%) with a Voc of 0.726 V, a Jsc of 13.65 mA cm^−2^, and an FF of 0.63. VS_2_ possesses low ionic conductivity as well as electrical conductivity, which was addressed by Zhuang et al. [168] by doping Ag into CoS. The authors concluded that doping Ag (5%) could substantially enhance conductivity and the electrochemical response to the redox reaction. The DSC device with a Ag-CoS CE showed a PCE of 8.35%, whereas a pristine Pt CE and CoS displayed 8.17% and 6.93% efficiency, respectively. Yet another CoS compound which was embedded in a graphene nanosheet (Gns) as a CoS-Gns CE produced a PCE of 7.28%, which was very close to that of a Pt CE. This was attributed to extension of the active surface area. Meanwhile, the doping of MoSx into a Co-Fe framework via solvothermal reaction was studied by Xu et al. [169]. The DSSC based on this framework offered a higher PCE of 9.63% than that of a Pt CE (8.23%). The 3D nanostructure with a bulky specific surface area exposing more edge boundary sites was responsible for this performance. Multiple compounds containing Co-Fe-S [170], CoFeS_2_ [171], and CoNi_2_S_4_ [172] as CEs in DSCs showed PCEs of 9.06% (Pt. 8.16%), 8.30% and 4.03% (Pt. 4.59%), respectively. A DSSC with a CoNi_2_S_4_-RGO nanohybrid as the CE exhibited a PCE efficiency of 9.22%. The active sites and photocatalytic performance of the CoNi_2_S_4_-RGO nanohybrid contributed to the DSSC’s performance [173]. Quaternary Gr based on Cu_2_ZnNiSe_4_–WO_3_ (G-CZNS@W) nanorods as the CE displayed an impressive PCE of 12.16%, superseding that of a Pt (4.07%) CE. This proves that a G-CZNS@W nanoscale hybrid framework could be an alternative CE for high-performance DSSCs [174].

A proper crystalline orientation is very important for a CE to govern the redox reaction of an electrolyte. Due to its high energy, as determined by CV and polarization quantification, the 001 plane of the CE crystal offers an improved catalytic reaction. Sulfides of iron show a variety of chemical formulas depending on the stoichiometry of Fe and S in the lattice. The presence of more Fe increases the catalytic active area for the FeS CE lattice. Wang et al. [175] synthesized a hexagonal FeS NS (2D) containing a high-energy 001 plane (FeS-HE-001) by the solution route using sublimed sulfur and FeSO_4_·7H_2_O. The DSC with the FeS-HE-001 CE achieved the highest PCE of 9.10%. The Pt CE device had a PCE of 7.73%, which was nearly 1.15% lower, measured under the same conditions, compared to that of the former one. Shukla and team [176] prepared FeS_2_ films by combustion of FeCl_3_ and CH_4_N_2_S in the absence of oxygen for DSSCs as a CE. The DSCs showed a high PCE of 8.0%, which was higher than that of a Pt CE in the same environment. The improved performance of the DSC was attributed to the higher redox reaction activity exhibited by FeS. Moreover, optical reflectivity might increase light absorption. A competitive PCE (6.47%) and device stability were achieved with FeS NRs as a CE by Zhang et al. [177]. FeS owns a higher number of electron–hole pairs and has p-type electrical conductivity. Moreover, Fe’s valence might also simplify the electron transfer between the CE and redox shuttle. The CE of an FeS_2_–carbon shell composite (FeS_2_/C) on carbon cloth (CC) offered a PCE of 8.15%, larger than that of a Pt CE (7.61%). Adhesion between CE components facilitated better stability as well [178]. The effect of the shape of FeS_2_ NCs as a CE was studied by Phuong et al. [179]. The cubic and spherical crystalline forms of the FeS_2_ NCs CE showed PCEs of 6.2% and 6.9%, respectively, while a Pt CE resulted in a PCE of 7% in the same *I*^−^/$I_{3}^{-}$ redox solution. Se, Te and other metals are well known for their advanced charge-transfer capabilities. Feng et al. [180] showed that Co_0.85_Se and Ni_0.85_Se as CEs exhibited PCEs of 9.40% (Pt 8.64%) and 8.32%, respectively. Tang and co-workers studied a series of metal selenides such as Co0.85Se, Ni_0.85_Se, Cu_0.5_Se, FeSe, and Ru_0.33_Se as highly reflective CEs for bifacial DSSCs. However, Co_0.85_Se and Ru_0.33_Se increased the PCE of the pristine Pt CE [181]. Moreover, selenides of Ni (8.69%), Co (7.30%) [76] and Nb (7.34%) also showed satisfactory performance [182,183,184]. Meanwhile, CoTe and NiTe_2_ CEs exhibited PCEs of 6.92% and 7.21%, respectively, nearly as high as the original Pt CE’s 7.04% in the same environment [185,186]. The metal phosphides, which could offer great fundamental redox reaction activity, possess comparatively bulky diffusion resistance, similar to metal nitrides and carbides. Though catalytically active, Ni_5_P_4_ and MoP are not sufficient to reduce I_3_^-^ [187]. Table 3 reports the CE preparation methods and related PCEs of alternative CEs in different DSSCs. Earth-abundant SnSe prepared via the electrodeposition technique was proposed as a CE by Zatirostami. The device offered a slightly lower PCE (4.9%) compared to a bulk Pt CE (5.1%) [188].

## 5. Limitations of Current Counter Electrodes (CEs) in DSSCs

Even though remarkable progress has been achieved, the performance of the present CEs in DSSCs is still restricted by problems relating to materials and interfaces. Although Pt is highly catalytic, it is expensive, rare and prone to corrosion in iodide/triiodide electrolytes, which prevents its widespread use. Carbon-based electrodes are cheap and conductive but have insufficient catalytic activity to enable efficient redox reactions. Transition metal sulfides, nitrides, carbides and oxides provide promising alternatives, but many of them show poor long-term stability, a short diffusion length or slow electron-transfer kinetics. Moreover, the adhesion between the CE and the substrate tends to fail when the electrode is exposed to thermal or chemical stress, which lowers its durability. It is still hard to attain the scalability and reproducibility of nanostructured CEs in industrial production. The main obstacle to replacing Pt is achieving a balance between cost, catalytic activity, conductivity and stability while at the same time making sure that high efficiency and long-term operational reliability are maintained in DSSCs.

## 6. Conclusions

This review looks at the most recent progress in the development of earth-abundant and low-cost CE materials as substitutes for Pt in DSSCs. As shown in the literature, in order to replace Pt, a careful balance must be achieved between electrocatalytic activity, surface area, chemical stability, electrical conductivity, charge-diffusion properties and manufacturing cost. Although carbon-based materials are very abundant, cheap and chemically stable, they generally have only limited intrinsic catalytic activity, and, therefore, it is necessary to carry out defect engineering and heteroatom doping or to combine them with metals and transition metal compounds in order to obtain performance similar to that of Pt. On the other hand, transition metal nitrides, carbides, sulfides and oxides have outstanding intrinsic activity, but it is necessary to carry out targeted optimization in order to improve their nanoscale morphology and diffusion pathways. Moreover, conductive polymers such as PEDOT offer a very practical solution for use in transparent and semitransparent DSSC designs because of their processability and flexibility.

The materials discussed in the literature show that Pt is still the standard catalyst (CE) because it has excellent intrinsic electrocatalytic activity, good electrical conductivity and proven long-term stability. Nevertheless, its high price and low abundance are still major obstacles to large-scale commercial use. Alternative materials such as carbon-based composites—especially those based on defect-engineered graphene and carbon nanotubes—provide one of the most promising combinations of power conversion efficiency, long-term stability, availability of the material and cost-effectiveness. Transition metal compounds have also shown catalytic performance comparable to that of platinum, but further improvements in long-term durability, scalable manufacturing, and reproducibility are still needed before they can be widely commercialized. Table 4 gives a summary of the potential catalyst (CE) materials for use in DSSCs.

## 7. Future Prospects

Future research into counter electrode materials should cease depending merely on compositional screening and instead should concentrate on the deliberate design of multi-component structures that achieve a balance between catalytic activity and long-term chemical stability. Researchers ought to work on optimizing ternary and quaternary transition metal complexes because these multi-element systems have particular electronic structures that increase the rate of charge transfer. When addressing morphology in the future, it is necessary to go further than just increasing the surface area; equal attention must be given to designing a strong physical adhesion to the substrate in order to avoid the delamination of the film when it is subjected to thermal stress. By overcoming these mass-transport and interfacial limitations through accurate surface engineering, platinum-free alternatives will be able to systematically attain the same efficiencies as the current benchmarks and thus offer a clear way toward scalable and commercially viable solar technology. Generally, the future development of CEs for DSSCs should be directed toward rationally designed composite materials that combine the high catalytic activity of transition metal compounds with the conductivity, chemical stability and low cost of carbon-based materials. If the new CEs are to progress from laboratory demonstrations to commercially viable DSSC technologies, advances will be required in the areas of interface engineering, defect engineering, nanostructure control, scalable fabrication methods and long-term stability assessment.

## Figures and Tables

**Table 1:** Summary of the principal characterization techniques used for CEs in DSSCs, the information obtained from each technique, and their significance for evaluating material properties and device performance.

Technique	Information Obtained	Importance of CE	References
Nyquist plots	Interfacial charge-transfer resistance and electrolyte diffusion.	Evaluates resistance, charge-transfer kinetics and electrocatalytic activity	[41]
XRD	Crystal structure, phase purity	Correlates crystallinity with catalytic activity	[42]
SEM/FESEM	Morphology, thickness	Reveals surface and film quality	[43,44]
EDX/EDS	Elemental composition, mapping	Confirms composition and dopant distribution	[43]
TEM/HRTEM	Nanostructure sizes, lattice fringes	Identifies nanoscale structure	[43,44]
AFM	Surface roughness	Relates topography to electrolyte contact	[42]
XPS	Chemical states and composition	Explains catalytic mechanisms	[42]
Raman	Carbon defects, graphitization	Important for graphene and carbon CEs	[42]
CV	Electrocatalytic activity, redox reversibility	Assesses catalytic performance	[45]
J–V measurement	Jsc, Voc, FF and PCE	Evaluates overall device performance	[16]

**Table 2:** Photovoltaic parameters of synthesized CEs.

Counter Electrodes	Voc/V	Jsc/mA cm^−2^	FF	PCE (%)	Reference
Gr/PANI-Cu^2+^	0.669	5.47	0.68	2.49	[137]
Gra/PANI	0.714	7.69	0.73	3.98	
Gr/PANI-Ni^2+^	0.737	8.56	0.63	4.00	
Gr/PANI-Co^2+^	0.740	9.19	0.68	4.65	
Gr/PANI-Mn^2+^	0.751	11.19	0.64	5.38	

**Table 3:** Summarizing the efficiencies of different Pt-free counter electrodes.

Alternative CE (Pt-Free)	CE Preparation Method	PCE (%)	Ref.
Carbon black–TiO_2_	Solid state	2.77	[88]
Graphene oxide–CNT	Spray coating	3.9	[189]
CoS-GNS	Spin coating	7.8	[108]
WC-C_MA_ (microwave-assisted)	Spray	6.85	[190]
WC-C_TA_ (two-step chemical activation)	Spray	7.32	[190]
Te–Mesoporous CNT	Electrospray	9.43	[95]
WO_3_-PANi composite	Electrodeposition	6.78	[191]
B-doped CNT (BCNTs)	Atmospheric pressure glow discharge (APGD)	7.28	[192]
SrTi_1−x_Co_x_O_3_ NPs/PEDOT-PSS	Hydrothermal	8.39	[193]
Dimethyl imidazolium iodide (DMII)-doped PEDOT nanofibers	Drop-casted	8.52	[194]
Ionic liquid (BVImI + LiI) modified MoS_2_	Electrodeposited	4.84	[166]
Potential-reversal MoS_2_	Potential-reversal electrodeposition	8.77	[195]
WS_2_–W_5_O_14_–MWCNTs	Hydrothermal	7.44	[196]
MWCNT-coated cotton fabric (M-AC/CNT CF),	Doctor blade method	6.06	[197]
MWCNT-coated polyester fabric (M-AC/CNT PF)		6.26	
MWCNT-coated linen fabric (M-AC/CNT LF)		5.85	
MoS_2_/Co_3_S_4_ with the Mo/Co ratio at 1:4	Hydrothermal	6.77	[198]
MoSe_2_/PANI composite	Spread	8.04	[199]
Ru–N-doped template-free mesoporous carbon (Ru–N-TMC)	Electrostatic spray	11.42	[200]
NiS@MoS_2_	Hydrothermal	8.58	[201]
Heterostructure MoS_2_@SnS_2_	Hydrothermal	7.53	[161]
poly (3, 4-phenylenedioxythiophene)	Electrochemical polymerization technique	5.97	[202]
PEDOT-Ni_2_P-3	Spin coating	7.14	[203]
PEDOT-Co_2_P-3		6.64	
CoNi_2_S_4_	Hydrothermal	4.03	[172]
Zn_0.76_Co_0.24_S		4.59	
NiCo_2_O_4_/rGO/PANI	Chemical precipitation	4.67	[204]
PEDOT/rGO-Al_2_O_3_	Electrochemical deposition	2.15	[205]
Polythiophene–molybdenum sulfide (PTh/MoS_2_)	Hydrothermal	1.36	[206]
Ammonium Lauryl Sulfate (ALS)-PANI	Doctor blade	4.54	[207]
PANi/9%GR	In situ polymerization	7.45%	[208]
Polyvinyl acetate (PVAc)/Graphene	In situ polymerization	4.57	[209]
GNP-Si_3_N_4_/PEDOT:PSS	Spin Coating	5.24	[210]
Mo_0.7_Fe_0.3_/rGO	Dry plasma reduction	5.44	[119]
S-rGO/MoS_2_	Spray	6.96	[117]
Ni-doped FeS_2_/rGO	Hydrothermal	7.60	[116]

**Table 4:** Comparative assessment of the principal CE materials discussed in this review with respect to catalytic activity, long-term stability, cost-effectiveness, scalability and overall potential for practical DSSC applications.

Counter Electrode	Catalytic Activity	Long-Term Stability	Cost-Effectiveness	Scalability	Overall Assessment	References
Platinum (Pt)	Excellent	Excellent	Poor	Moderate	Benchmark material with the highest catalytic performance but limited by cost and rare.	[47]
Carbon black/Graphite	Moderate	Good	Excellent	Excellent	Low-cost alternative is suitable for large-area devices but requires performance enhancement.	[79,80]
Graphene and CNTs	Good–Excellent (after defect engineering or doping)	Excellent	Excellent	Good	Highly promising owing to excellent conductivity, stability, and tunable catalytic activity.	[102,211]
Carbon-based composites	Excellent	Excellent	Excellent	Good	One of the most promising alternatives, combining high efficiency with low cost and durability.	[212]
Transition metal carbides/nitrides/sulfides/oxides	Excellent	Good	Good	Moderate	Catalytic activity approaching Pt; further improvements in durability and scalable synthesis are required.	[144,151]
Conductive polymers (PEDOT, PANI)	Good	Moderate	Excellent	Excellent	Attractive for flexible and transparent DSSCs, but long-term stability remains challenging.	[136,139]

## Data Availability

No new data were created or analyzed in this study. Data sharing is not applicable to this article.
